# Cyber-Pedagogy to the Rescue: Creating Effective Online Programming for Students and Trainees During the Pandemic

**DOI:** 10.1093/geroni/igab046.1052

**Published:** 2021-12-17

**Authors:** Kara Dassel

**Affiliations:** University of Utah, Salt Lake City, Utah, United States

## Abstract

COVID-19 upended in-person educational programming in areas such as classroom instruction within academic institutions, engagement of adult learners, and training of direct care workers. In-person educational offerings were forced, as a result of health restrictions, to pivot into either asynchronous or synchronous web-based instruction. This panel discussion will discuss lessons-learned in cyber-pedagogy in three areas: 1) Faculty Consultation: Faculty who teach online regularly and have completed training in quality online educational practices are experts who were called upon to assist others with transitioning courses to an online format. This presentation outlines the ways in which certified online instructors, at one academic center, tutored and assisted faculty in the health sciences with online instruction. 2) Adult Learning: The rapid transition to online learning has implications for adult learners pertaining to accessibility, diverse learning and technology abilities, and course and peer engagement. This presentation will explore strategies that faculty can utilize to offer adult learners differentiated learning and engagement opportunities. This discussion will also highlight the nexus among these pedagogical strategies and the Age-Friendly University Global Network, providing guidance for how universities can connect online learning methods to the Age-Friendly University principles. 3) Workforce Training: The direct care workforce employed in community-based services and support programs and long-term care settings tend to receive little or no training in geriatric care. This presentation will discuss how educational trainings offered through a Geriatric Workforce Enhancement Program transitioned onto web-based platforms in order to accommodate these ongoing educational needs throughout the pandemic.

